# Quality Control Method and Device for Producing Agarose Micropads

**DOI:** 10.17912/micropub.biology.001081

**Published:** 2024-01-26

**Authors:** Martin Vo, Lance Kuo-Esser, Mauricio Dominguez, Julia Driggers, Kennedy Kuchinski, Sheny Perez, Taylor Luck, The Tran, Thang Tran, David Gerberry, Hanna Wetzel, Wilber Escorcia

**Affiliations:** 1 Lake Erie College of Osteopathic Medicine, Erie, Pennsylvania, United States; 2 Chemistry Department, Xavier University, Cincinnati, Ohio, United States; 3 Biology Department, Xavier University, Cincinnati, Ohio, United States; 4 Mathematics Department, Xavier University, Cincinnati, Ohio, United States

## Abstract

In brightfield and fluorescence microscopy, capturing images that show well-focused and immobile microorganisms can be challenging. An agarose-based gel pad reduces the variability of results, especially in conditions like uneven specimen staging, variable fluid dynamics, and Brownian motion that plague conventional wet mount setups. To correct these discrepancies during image acquisition, we analyzed three micropad preparation setups. We tested the quality and consistency of pads and images resulting from each setup. Our examination reveals that improved gel pad flatness is associated with better image quality. Moreover, we observe increased consistency in gel pad construction connected to the use of a 3D-printed setup. These findings highlight the technical benefits arising from incorporating micropad-generating platforms that increase the consistency of results in imaging pipelines. Additionally, our use of a quantitative approach to examine pad flatness suggests its inclusion in quality control pipelines to reduce variation in gel pad construction and image quality over time and between investigators. Finally, our use of a 3D-printed setup coupled with a quantitative downstream routine suggests their application in microscopy experiments that involve model organisms relevant to human health and disease.

**Figure 1. Optimizing agarose gel micropad production and imaging with a 3D printed device and QC method:**
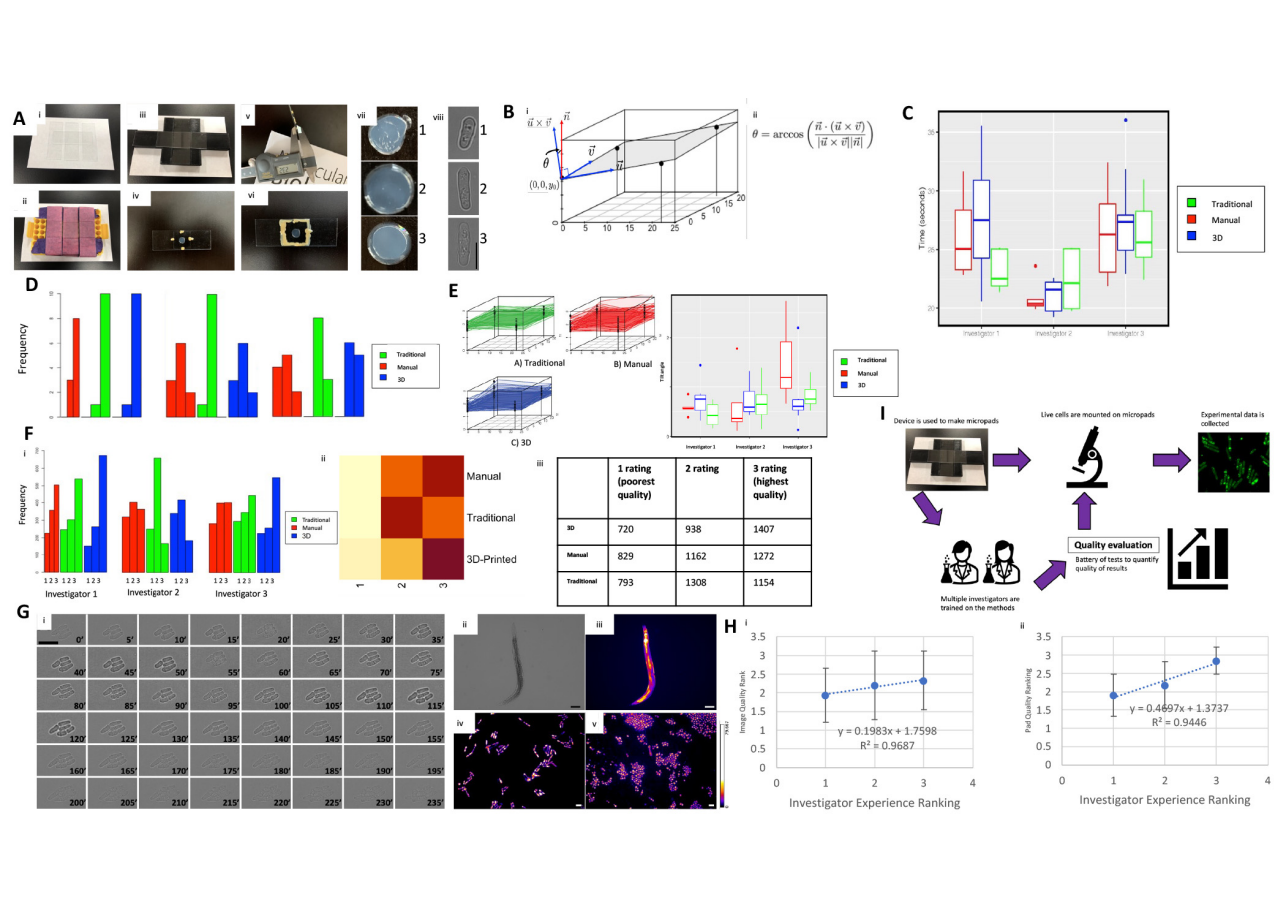
**A.** Agarose gel pad preparation methods and measuring of tilt angle parameters **.** i) a manual setup, made of two slides laid in parallel, ii) a traditional setup made of a pipette-tip rack and lab tape, iii) a 3D printed micropad maker, iv) a micropad with a coverslip temporarily fixed on the sides with VALAP, v) The digital caliper used to measure the height of each corner of the coverslip, and vi) a micropad with a coverslip sealed on all sides with VALAP, vii) examples of micropads of differing qualities ranked 1-3, where 1 is unusable and 3 is ideal, and viii) examples of imaged cells with different focus qualities ranked 1-3, with 1 being out-of-focus and 3 being ideally focused. Scale bar represents 5 µm in length. **B.** i) The angle of tilt of the coverslip was calculated using regression and ii) Equation 1. **C.** The time taken to produce agarose pads. The time taken by three different investigators to make a single agarose pad slide using the manual method (red), a traditional method (green), and the 3D device (blue) (n=11 slides per investigator per method). Investigator 2 was significantly faster than both Investigator 1 and Investigator 3, but no significant difference was detected between methods. **D.** Micropad quality ratings between investigators and production methods. Agarose pads made using the manual method (red), a traditional method (green), and the 3D device (blue) (n=11 slides per investigator per method) were rated on a 1-3 scale, where 1 is an unusable pad and 3 is an ideal pad. Quality ratings differed significantly between investigators, but not between methods. **E.** The angle of tilt of the coverslip over the agarose pads made by three different investigators to generate a single agarose pad slide using the manual method (red), a traditional method (green), and the 3D device (blue) (n=11 slides per investigator per method). Tilt angles differ significantly between investigators, but not production methods. **F.** Image quality rankings between methods and investigators. A rating of 1 indicates an unfocused image, while a rating of 3 was assigned to ideal images. i) Shows the frequency of each rating given to brightfield microscopy images taken on pads made using the manual method (red), a traditional method (green), and the 3D device (blue). ii) shows a heat map of the different ratings across investigators using all 3 methods. Darker spots on the map indicate higher frequencies of a given rating while lighter spots indicate smaller frequencies. Eleven micropads (n=11) per investigator and per method were used. Three images were taken per micropad, and 33 cells per image were rated on a 1-3 scale. iii) shows a table representation of the total amount of pictures that were taken on each of their respective graded micropads. **G.** Image Focus Quality in *S. pombe, C. elegans,* and *S. cerevisiae* . i) A frame-by-frame, live-cell microscopy montage of fission yeast cell duplication in increments of 5 minutes up to 4 hours. ii-v) Brightfield and GFP fluorescence images of *C. elegans* strain RW1596 ( *myo-3* ( st386 ) V; stEx30 ). Scale bar represents 100 µm in length. iv-v) Fluorescence imaging of fission and budding yeast, respectively, whose lipid droplets were stained with Nile Red. Scale bar represents 10 µm in length. For fluorescence images, a fire look-up table (LUT) was used to generate profiles where brightness is linked to signal intensity. **H.** Comparison of image and pad quality across investigator experience. The experience rank of the investigator correlates with both i) the quality ratings of the microscopy images (n=1089 cells per investigator) and ii) agarose pad quality rating (n=33 slides per investigator). **I.** Micropad production workflow in microscopy image acquisition experiments. Starting with the 3D device, micropads are made and then organisms are transferred onto the pad. Afterward, images are taken with a microscope. Each part of this workflow provides a checkpoint to check the quality of each step.

## Description

*Introduction*

Capturing images of microorganisms in liquid-mounted slides is challenging because of constant mobility in the medium, cellular aggregation, and localization at different focus planes. Overcoming the physical limitations imposed by liquid surface tension and Brownian motion is facilitated by a specimen-staging platform. A microgel pad (i *.e.,* micropad) generated with molten agarose sandwiched between two microscope slides provides a firm yet versatile structure that addresses the primary shortcomings associated with liquid-mounted slides (Escorcia et al *.* , 2019). Consistent quality production of micropads is a vital step in cellular and molecular biology research.

To test production quality, we produced three micropad-fabrication setups. We employed a parallel-slides setup (manual method), the simplest way of generating a micropad ( Figure 1Ai ). This involved placing a pair of microscope slide stacks (2 slides tall) surrounding a middle slide to create a groove in which molten agarose is dispensed and shaped into a circular micropad (an agarose pancake). A perpendicular slide was used to compress the gel to its final circumference and thickness. However, this method relies heavily on investigator experience to produce quality results. In addition to the manual method, we explored two alternative methods: the lab-tape setup (traditional method) and the 3D-printed setup (3D method). The lab-tape setup recapitulates the manual design but creates layers of lab tape instead of microscope slides to create the two stacks with the central groove where agarose is dispensed ( Figure 1Aii ). This method allows for a more dynamic way to adjust the heights on the stacks. On the other hand, the 3D-printed setup was designed to generate a similar concept where there are two stacks with the central groove in the middle. Side handles and a spot for the fingers to naturally press over the slide to improve stability was implemented as well. The design was carried out using TinkerCAD and fabricated with a MakerBot 3D printer, providing product consistency and experimental replicability ( Figure 1Aiii ). This method allows for the most precise, accurate, and consistent way of making micro pads as the device measurements is printed the same way each time. Because the design is digitally generated, additional features can also be added and printed in the future as the experimenter sees fit.

We compared outcomes from these three setups to determine micropad and image quality as well as investigator efficiency ( Figure 1C -D). Little work has been done to develop methods that quantitatively assess the quality of micropads (Priest et al *.* , 2017; Rivera and Schvarzstein, 2018). Quality control (QC) is an important step in the scientific process, and quantitative metrics of quality are key to robust QC protocols. A battery of tests to determine the quality of micropads between investigators, production methods, and fabrication times would help certify new users and maintain quality production across experiments. Therefore, and since all methods result in an agarose gel pad interposed between a microscope slide and a coverslip ( Figure 1Aiv -vi), we examined the physical characteristics and thickness of pads to determine their utility in microscope imaging ( Figure 1Av ). Parameters such as circularity, air bubbles, cracks, and uneven surfaces were used as scoring guidelines to compare each method ( Figure 1Avii -viii). Additionally, we measured the height of each micropad corner delimited by the coverslip to extrapolate micropad flatness ( Figure 1Av ).

For each micropad, we used four corner points to calculate the angle of tilt. This metric allowed us to determine micropad flatness and to compare production quality among the three methods. Because flatness is associated with specimen focus, we implemented a quantitative approach to provide baseline quality control profiles to assess the effectiveness of each method and investigator. Our examination of three micropad preparation methods suggests a useful QC routine that can be applied to train novice investigators and ensure replicability across microscopy experiments and labs. The feedback generated by calculating micropad flatness allows researchers to correct technical errors, fine-tune imaging outcomes, and increase experimental efficiency. This is particularly important in microscopy applications such as live-cell microscopy, where prolonged image acquisition is dependent on micropad quality and integrity (Escorcia et al., 2019; Escorcia et al *.* , 2021).

*Results*

*Quality and Efficiency of Micropad Production*

Preparing slides is a limiting step in most microscopy pipelines. Though micropads can be produced by the three methods we examined, only high-quality products lead to reliable and consistent imaging data. Therefore, we asked how investigator experience and method type contributed to micropad quality. Both the speed and quality of production varied significantly between investigators, but not between production methods ( Figure 1C -D). The experienced investigator (investigator 1) produced on average significantly higher quality slides than the two less seasoned researchers (investigator 2 and 3), with an average rating of 2.85 relative to 1.91 and 2.18, respectively, for the other investigators. Investigator 2 was significantly faster than their counterparts (21.05 seconds, compared to 25.18 and 26.52 seconds, p<0.0001). However, this researcher also produced the lowest quality slides. There was no significant difference in the quality rating of slides between methods (p=0.153), but there was a significant difference between investigators (p<0.0001). These results indicate a useful mechanism to differentiate researchers based on their efficiency in constructing quality micropads using different methods.

*Angle of Tilt and Micropad Flatness*

Micropad unevenness contributes to out-of-focus, low-quality images. Thus, we asked whether the calculated tilt angle resulting from pads was impacted by production method, and if differences in flatness would correlate with micropad quality. A tilt angle of zero represents a completely flat plane. We observed that the manual method produced pads with higher, more variable tilts (i *.e.,* higher “ *dishevelness* ”) as compared to the 3D and traditional methods ( Figure 1E ). However, while the difference is moderate, it does not show statistical significance (p=0.069). On the other hand, significant differences were detected between investigators (p=0.016), which highlights the variability introduced to micropad production between different researchers. These results suggest that tilt angle can be used as a proxy to gauge flatness in micropad production and serve as a guide to improve product quality.

*Microscope Image Focus and Quality*

Micropad fabrication is a step upstream of image acquisition. As a result, alterations to micropad quality affect image characteristics that are important for data integrity. Since we observed that image focusing is closely connected to micropad flatness, we asked if there were differences associated with pad preparation setups and investigator experience. Image quality ratings varied significantly between both investigators and the production method (p<0.0001). Our data reveal that relative to other setups, the manual method is linked to lower quality images regardless of investigator experience ( Figure 1F ), with the highest frequency of images rated 1 occurring in this group. The 3D method produced the highest number of images that achieved a rating of 3, indicating that it overall produces the highest quality images of the 3 methods. The traditional and 3D setups show outcomes that are dependent on the length of technical training ( Figure 1F ). These data, therefore, imply a close relationship between pad preparation method and pad quality that can be modified in microscopy pipelines to enhance specimen staging. This is particularly important when prolonged image capture ( Figure 1Gi ), live fluorescence imaging ( Figure 1Gii -iii), or quantitative fluorescence methods ( Figure 1Giv -v) are employed.

*Micropad Quality and Investigator Experience*

As with most technical expertise, generating micropads suitable for data acquisition relies on skill and extensive familiarity with the setup used. Consequently, we asked if there was a correlation between investigator experience as well as micropad and image quality. We observed that seasoned investigators are linked to better micropad quality and thus better microscope images ( Figure 1H ). Although this observation is intuitive, our quantitative quality control approach provides useful data that confirms the expected trend. This indicates its applicability to gauge expertise progression gained through gradual training.

*Conclusion*

In this study, we aimed to establish a method by which we could implement quality control steps during the fabrication of agarose gel micropads. Building a reliable micropad increases the probability of quality data acquisition. Investing time and effort into properly staging specimens results in high-quality imaging, which is key to achieving reliable, accurate data further down the pipeline. Therefore, it is critical for investigators to examine each point in microslide preparation that contributes to consistent imaging outcomes.

Our findings reveal that quantifying micropad construction quality and efficiency serves to assess the staging preparation capacity of investigators with different experience levels ( Figure 1I ). The predictive potential of this approach will benefit from including a larger sample size of researchers and specimens to analyze in the future. Moreover, calculating tilt angle offers plane projections that graphically illustrate and statistically indicate micropad flatness. While often overlooked in practice, this metric offers a useful consideration when selecting the most appropriate setup or best-qualified personnel to prepare slides for imaging. These metrics could also be used to develop routines to certify new lab members in micropad production before allowing their products to be used in key experiments. Connecting investigator skills with micropad and image quality allows researchers to not only implement quality control checkpoints in an experiment but also provide constructive feedback for to both training inexperienced and season microscopy researchers.

Micropads represent a fundamental early step in the pipeline to quality imaging data. We present a robust battery of tests that can be used to assess micropad quality. We also provide evidence for the use of a 3D-printed device to improve reproducibility. Together, these methods will lead to improved data for experiments that rely on dependable staging of the specimen.

## Methods

*Parallel-Slides Setup (Manual)*

We implemented a straightforward method for micropad generation using a parallel-slides setup. This involved placing a pair of microscope slide stacks (2 slides tall) around a middle slide, creating a groove for dispensing, and shaping molten agarose into a micropad ( Figure 1Ai ). A perpendicular slide is used to compress the gel to its final circumference and thickness. Although minimal effort and materials are required to build this setup, it relies on extensive technical mastery to produce micropads of consistent thickness and flatness. In this work, we refer to this setup as *manual* , which denotes a high level of dexterity needed to produce quality slides.

*Lab-Tape Setup (Traditional)*

To enhance compression control and reusability across experiments, we introduced the lab-tape setup. Mimicking the manual design, this approach utilizes lab tape instead of microscope slides to create the central groove for agarose dispensing ( Figure 1Aii ). The stacking of lab tape affords researchers the flexibility to adjust micropad width and flatness according to experimental needs. Despite greater control over micropad fabrication relative to the manual setup, the traditional version lacks dimension consistency across labs and investigators. We decided to label this setup as *traditional* because its basic design originates from a traditional yeast genetics lab, the Forsburg Lab.

*3D Printed Setup (3D)*

For improved product consistency and experimental replicability, we designed a micropad-maker using TinkerCAD and fabricated it with a MakerBot 3D printer ( Figure 1Aiii ). Our device was built from acrylonitrile butadiene styrene (ABS) filament, features a cross design for loading stability, and has a central groove (1.5 mm thick, 0.5 mm wide) that fits standard microscope slides (VWR, 16004-422) ( Figure 1Aiii ). Because the TinkerCAD website allows investigators to export the design to different printers, the micropad-maker setup provides product consistency and experimental replicability that can be easily recapitulated between laboratories and investigators. We named this setup as *3D* to highlight its printed origin. The image schematic and printing instructions for the device can be found at this GitHub link: **https://github.com/dhakxls/MicroPad** .

*Micropad Preparation*

Micropads were produced as previously reported (Escorcia et al., 2019; Escorcia et al., 2021; Vo et al., 2022) with minor modifications. Briefly, we aliquoted 50 µL of molten agarose onto a central groove with a bottom slide. We then used a perpendicular slide placed on top to compress the gel into a circular shape and allowed the micropad to solidify. Specimens were loaded onto the finished micropad, excess medium was removed by inverting the slide setup and blotting the pad surface on a lint-free paper towel. A coverslip was then affixed on top and rotated with the finger twice to disperse cells into a monolayer. To keep the coverslip in place, we used a sealant containing a 1:1:1 weight ratio of Vaseline, lanolin, and Paraffin (VALAP). When heated up, VALAP is liquid and can be applied to the edges of the coverslip with a wooden stick. This must be done quickly to prevent untimely solidification. Once sealed, the specimen must equilibrate to the microscope environment for 15 minutes and can be subsequently imaged for up to 8 hours without substantial evaporation or shifting.

*Assessment of Agarose Pad Production*

Three investigators with differing amounts of experience were trained on all three production methods to make agarose pads. Two practice rounds were carried out before testing was initiated. Eleven slides were prepared per method for a total of thirty-three slides per investigator. The pads were scored on a scale of 1-3, where 3 was an ideal agarose pad (circular, level, and without bubbles or cracks), 2 was usable but slightly uneven or contained some bubbles or cracks, and 1 was unusable for data collection ( Figure 1Avii ). A similar scoring scheme was used to qualify the focus and quality of images resulting from each method. A score of 3 indicated a fully focused cell, while 1 represented a mostly blurry image ( Figure 1Aviii ). Moreover, we recorded the time each investigator took to generate a micropad to gauge production efficiency ( Figure 1Aiii ). Finally, to determine micropad flatness, the height of each corner of the coverslip per micropad was measured in micrometers (1-0.01 µm) with a digital caliper (Fowler sylvac, model S 235) ( Figure 1Av ). To ensure corner measurements were consistent, we secured the four edges of the coverslip with VALAP dots, which prevented unwarranted movement during caliper manipulation at the corners ( Figure 1Aiv -v).

*Quantification of the tilt angle of the coverslip*

Each corner measurement represents a point in 3-dimensional space. As a plane is completely determined by three points, the four measurements at each corner of the coverslip over-determine the plane. In other words, the four points will not lie on the same plane due to unavoidable measurement error. To address this, regression was used to find the plane that fit the four corners with the least square error ( Figure 1B ). The regression planes with *R* ^2^ < 0.70 were dropped as they indicated thickness measurements that were inconsistent with a plane due to measurement error. The regression plane can be described by the equation *z = y _0_ + ax + by,* where *a* and *b* represent the slopes of the plane in the direction of vectors *u* and *v* , respectively, and the height in the lower left corner is *y _0_* ( *i.e.,* indicated by the point *(0, 0, y _0_* ) in Figure 1B ) *.* To find the tilt angle of each plane, which we denoted 𝜃, we note that the points (1, 0, *y _0_ + a)* and (0, 1, *y _0_ + b)* exist on the regression plane and that a vector that is perpendicular to the plane can be found by taking the cross product of the vectors *u* = *(0, 0, y _0_* ) - (1, 0, *y _0_* + a) and *v* = (0, 0, *y _0_ ) - (0, 1, y _0_ + b)* . The tilt angle is then the angle between this vector and the standard normal vector ( *i.e* ., “ *straight up* ” vector denoted *n* in Figure 1B ) which can be calculated using the following equation in Figure 1Bii .

Micropad flatness was examined by comparing the tilt angle differences produced with each pad preparation method, where a tilt angle of zero represents a totally flat plane. We also illustrated the capacity of each setup to produce flat micropads by showing the extent of stack “ *dishevelness* ” resulting from fabrication. With this metric, researchers can keep track of the consistency with which micropads are generated by investigator experience and method used.

*Preparation and Image Acquisition of Worms*

*Caenorhabditis elegans* were cultivated and manipulated as previously reported (Escorcia et al *.* , 2018; Hammerquist et al *.* , 2021). Briefly, animals were grown on nematode growth medium (NGM) plates seeded with late-log OP50 *E. coli* at 20°C until the early L4 stage. Worms were washed off the plates with M9 into 1.5 mL microfuge tubes. The worms were then centrifuged at 560 x *g* for 1 minute, and the supernatant was removed. This was repeated until the *E. coli* was cleared from the suspension. Paralysis was induced with 1 mL of 25 mM sodium azide (NaN _3_ ) which was added to each tube. 5 µL of worm suspension was dispensed onto a fresh agarose pad, and a coverslip was placed on top. The pad was rotated to ensure an evenly distributed sample and a monolayer of the organisms. Imaging was conducted at 5x magnification with multiple worms per field of view, followed by 10x magnification for higher-resolution images. Nematode strains N2 and RW1596 ( myo-3 ( st386 ) V; stEx30 ) were acquired from the Caenorhabditis Genetics Center (CGC) at the University of Minnesota and were used in fluorescence microscopy images.

*Fission Yeast Growth and Culture*

The techniques described, culture conditions, and media were followed as previously described (Sabatinos & Forsburg, 2010) . Liquid medium experiments used yeast extract with supplements (YES 225, 2011-300; Sunrise Science Products). Subculturing involved transferring cells from a 3 mL YES starter to a 3 mL YES proliferation tube and growing them at 32 ^o^ C in an incubator shaker until reaching OD _abs595_ 0.3-0.6. Cells were then harvested and washed three times in YES and prepared for microscope acquisition as reported previously (Escorcia et al., 2019) . For live-cell microscopy, cells were placed on 2% agarose micropads made with liquid EMM plus supplements as detailed elsewhere (Escorcia et al., 2019) and imaged at room temperature (22 ^o^ C). Strains FY527 (wildtype) and MP218 ( *cut6 -621* ) were acquired from the Susan L. Forsburg and Peter Espenshade Labs, respectively, and were used in live-cell microscopy experiments.

*Bright field Microscope Image Acquisition*

Imaging was performed following the methods detailed in Escorcia et al., (2017, 2019, 2021) with minor alterations. Cells were staged at room temperature (22°C) using an inverted BZ-X800E Keyence microscope equipped with 40x (0.95 NA) and 60x (1.40 NA) oil immersion Plan Apochromat objective lens (BZ-PA60), a 3.7 W LED light source, an 8-bit monochrome CCD camera (2.3 million pixels), and BZ-H4A BZ-X800E Analyzer software. TIF files were saved for subsequent data analysis. For live cell microscopy experiments, images were automatically taken every five minutes for a period of four hours. Micrograph panels were created using Fiji (Ferreira and Rasband, 2023) as detailed elsewhere (Escorcia et al *.,* 2019).

*Data Analysis and Statistics*

For each method analyzed, each investigator generated 11 micropads. Three images were taken for each pad in different locations across the pad. For each image, 33 cells were rated for quality where a rating of 3 was an optimally focused image, 2 was an intermediate quality, and 1 was an unfocused image ( Figure 1H ). This yielded a total of 9846 cells that were analyzed and ranked. Slide quality ratings and production times were statistically compared between investigators and between devices using two separate one-way ANOVAS with Tukey *post-hoc* tests. The frequency of different ratings of pads and images were compared using a Fisher’s Exact test or Chi-squared tests when appropriate. All statistics and figures were done in R script (R Core Team, 2021) , GraphPad Prism, or Microsoft Excel. We provide all R scripts in the following GitHub link: **https://github.com/dhakxls/MicroPad** .
